# Intra-Personal and Inter-Personal Kinetic Synergies During Jumping

**DOI:** 10.1515/hukin-2015-0110

**Published:** 2015-12-30

**Authors:** Kajetan Slomka, Grzegorz Juras, Grzegorz Sobota, Mariusz Furmanek, Marian Rzepko, Mark L. Latash

**Affiliations:** 1 Department of Human Motor Behavior, The Jerzy Kukuczka Academy of Physical Education, Katowice, Poland; 2 Department of Biomechanics, The Jerzy Kukuczka Academy of Physical Education, Katowice, Poland; 3 Department of Physical Education, University of Rzeszow, Rzeszow, Poland; 4 Department of Kinesiology, The Pennsylvania State University, State College, Pennsylvania, USA

**Keywords:** jumping, synergy, anticipatory synergy adjustments, inter-personal interactions

## Abstract

We explored synergies between two legs and two subjects during preparation for a long jump into a target. Synergies were expected during one-person jumping. No such synergies were expected between two persons jumping in parallel without additional contact, while synergies were expected to emerge with haptic contact and become stronger with strong mechanical contact. Subjects performed jumps either alone (each foot standing on a separate force platform) or in dyads (parallel to each other, each person standing on a separate force platform) without any contact, with haptic contact, and with strong coupling. Strong negative correlations between pairs of force variables (strong synergies) were seen in the vertical force in one-person jumps and weaker synergies in two-person jumps with the strong contact. For other force variables, only weak synergies were present in one-person jumps and no negative correlations between pairs of force variable for two-person jumps. Pairs of moment variables from the two force platforms at steady state showed positive correlations, which were strong in one-person jumps and weaker, but still significant, in two-person jumps with the haptic and strong contact. Anticipatory synergy adjustments prior to action initiation were observed in one-person trials only. We interpret the different results for the force and moment variables at steady state as reflections of postural sway.

## Introduction

The word synergy has been used in at least three meanings in movement science. In clinical literature, synergy frequently means stereotypical patterns of muscle activation (for example, after a cortical stroke) that interfere with voluntary movements (Bobath, 1978; Dewald et al., 1995). In motor control literature, in line with the traditions of Bernstein (1967) synergy is defined as a set of variables (for example, muscle activations, joint torques, or joint trajectories) that show parallel scaling with changes in task parameters or with time during action execution (d’Avella et al., 2003; Ivanenko et al., 2004; Ting and Macpherson, 2005). According to this definition, the main purpose of synergy is to decrease the number of variables manipulated by the neural controller. This definition is directly linked to the famous problems of motor redundancy, the excess of elemental variables at any level of description of the neuromotor system. The problem of motor redundancy has recently been recast as the principle of abundance (Gelfand and Latash, 1998; Latash, 2012). According to this principle, the apparent excess of elemental variables is not a computational problem, but a powerful apparatus that allows organizing stability of important performance variables in a task-specific way (Schoner, 1995). Within this approach, synergy is defined as co-variation of elemental variables that stabilizes (reduces inter-trial variance of) an important performance variable (Latash et al., 2007). Within this paper, we accept the last definition of synergy.

Synergies stabilizing a variety of performance variables within a variety of tasks performed by a variety of populations have been documented (Latash, 2008; Latash et al., 2007). Most of these studies explored synergies in tasks performed by one person only. Studies of motor coordination in groups of two or more persons required participants to perform individual motor tasks while watching each other (Fine and Amazeen, 2011; Fine et al., 2013; Schmidt et al., 1990), talking to each other (Shockley et al., 2002; Stoffregen et al., 2009), or having haptic contact (van der Wel et al., 2011). Several of those studies have reported that participants in those groups exhibit patterns of behavior similar to those, which would be seen when one person coordinates multiple limbs (Fine and Amazeen, 2011; Fine et al., 2013; Schmidt et al., 1990). In particular, stabilization of a relative phase during two-limb motion has been reported in both one-person and two-person tasks and explored using a method based on the UCM hypothesis (Black et al., 2007; Riley et al., 2011).

In this study, we explored a whole-body task and performed analysis at the level of kinetic variables. This approach has been motivated by the fact that kinetic synergies in the lower extremities have been explored only for a handful of single-person tasks (Robert et al., 2008; Sarabon et al., 2013; Yen et al., 2009). Our aim was to discover whether two-person synergies stabilizing jointly produced performance variables could be seen in the presence of visual feedback only, haptic contact, and strong mechanical contact. We also explored whether subject pairs would be able to show anticipatory synergy adjustments (ASAs), a feed-forward drop in the index of synergy stabilizing a performance variable prior to the initiation of a quick action (Klous et al., 2011; Olafsdottir et al., 2005).

The following hypotheses were tested. Hypothesis-1: During one-person tasks, synergies between force and moment variables produced by each foot would stabilize their resultant values during a steady state prior to the jump initiation (Sarabon et al., 2013). Hypothesis-2: Such synergies would be weak or absent between two persons jumping in parallel without additional contact, they would emerge with haptic contact and become stronger with “strong contact”. This hypothesis was motivated by models of synergies that involve either short-latency feedback loops within a single central nervous system (Latash et al., 2005) or sensory feedback on a jointly produced variable (Martin et al., 2009; Todorov and Jordan, 2002). Hypothesis-3: ASAs would be seen in one-person tasks and in two-person tasks with haptic or “strong” coupling, but not in two-person tasks performed under visual contact only. By definition, ASAs happen during steady-state task performance. Based on Hypothesis-2, no synergies are expected during a steady state under a visual contact condition; hence, no ASAs are expected.

To test the hypotheses, we quantified force and moment stabilizing synergies between the two feet (in one-person tasks) and between the two persons (in two-person tasks) during preparation to a long jump to a visual target from standing posture. The task was performed by one person alone and by two persons performing the task synchronously. In the latter condition, we varied coupling between the two performers, from visual coupling only, to haptic coupling (holding a coupling object with precision grip), and to “strong coupling” (placing the hand over the other person’s shoulder).

## Material and Methods

### Subjects

Twenty male subjects took part in the experiment. They were recruited from the students of the physical education course. Their average age, body mass and height were 22.5 ± 2.3 years, 73.3 ± 6.6 kg, and 179.3 ± 6.3 cm (mean ± SD), respectively. Subjects were matched into pairs by height. All subjects did not report any muscle or skeletal disorders and their fitness level was high. Prior to the experiment, subjects signed an informed consent form. The experimental procedures used in this study were in accordance with the Declaration of Helsinki and were approved by the Jerzy Kukuczka Academy of Physical Education Institutional Review Board.

### Apparatus

Two force platforms (AMTI, Accugait, USA) were placed side-by-side. The platforms sampling frequency was 100 Hz. AMTI Netforce software was used to register ground reaction forces (*F_X_*, *F_Y_*, *F_Z_*) and moments (*M_X_*, *M_Y_*) during the experiment, where *X*, *Y* and *Z* are medio-lateral, anterio-posterior and vertical axes respectively.

### Procedure

After a short warm-up, the matched subjects individually executed three standing maximal-distance long jumps with their hands clasped behind their backs. There was a short interval between the jumps to allow time for assuming a starting position for the next jump. These results were averaged for the pair (across six jumps) and 50% of this value was taken as the target distance for this particular pair of subjects. Next, the subjects performed a series of jumps to the target indicated by a line drawn on the floor. They were instructed to land with their heels at the line.

Four series of jumps were performed by all the subjects in a random order. In the first condition (C1) each subject within a pair jumped independently. They started in the standing position, with each foot placed on one of the platforms and with the arms clasped behind their backs. A comfortable foot position was selected with the big toes aligned with a line drawn 10 cm from the platform anterior border. The foot position was marked on the platform and reproduced across all the trials. The first 3–4 trials were used to acquaint the subject with the task. During each trial, the subject was instructed to signal his readiness for the trial and then to perform a jump at a self-selected time about 2–3 s later. There was no time pressure, and the subjects were instructed to focus on jump accuracy. We used the pre-trigger function in *Netforce* software to record 4 s of data during the steady state before each jump.

In the other three conditions subjects jumped together in parallel from separate platforms. In condition C2, there was no contact between the subjects and they held their arms behind their backs as in C1. In condition C3, the two subjects held a pencil-like wooden stick, one subject with the right hand and the other subject with the left hand, to provide haptic contact. The object-holding arm was held along the subject’s side with the elbow joint bent at 90°, the hand supinated and wrist extended. The object was held with the index finger and the thumb. In condition C4, the subjects placed their hands on the partner’s farther shoulder. The free hand in C3 and C4 was placed behind the subject’s belt. In each condition, subjects executed 5 familiarization jumps followed by 20 consecutive jumps. There was a 15 s break between jumps; this time was enough to assume the starting position for the next trial. All the jumps were executed barefoot.

### Data analysis

The data were processed with the Matlab software package. The raw data were low-pass filtered with the 7 Hz, 4^th^-order zero-lag Butterworth filter. The force values were normalized by body weight and moments were normalized to the product of subject’s weight by foot length.

### Identification of movement initiation time

In each trial, all three force components (*F_X_*, *F_Y_* and *F_Z_*) were analyzed, for each platform separately, to find the earliest time when a change in each of the force components was over 3 standard deviations from the average value computed over the first 2 s of quiet standing. The earliest of the three time values was taken as the movement initiation time (T_0_), and all the trials were aligned by T_0_. The data within the time interval between −500 ms before T_0_ and +1000 ms after T_0_ were further analyzed.

### Analysis of co-variation

Linear regressions between the matched pairs of force and moment variables recorded by the two platforms in consecutive trials were calculated using the data from 20 jumps for each condition and for each time sample. This was done for each force component (*F_X1_* vs. *F_X2_*, *F_Y1_* vs. *F_Y2_*, and *F_Z1_* vs. *F_Z2_*) and each moment component (*M_X1_* vs. *M_X2_* and *M_Y1_* vs. *M_Y2_*) separately. The correlation coefficients (*R*) and regression coefficients (*B*) were computed: Y = a + *B*X. The *R* values were normalized using Fisher’s Z-transform for further statistical analysis resulting in *R_Z_* values. We used linear regression because, for all pairs of variables, we expected them to covary negatively corresponding to synergies stabilizing their sum (the resultant variable). For such analyses, computing linear regression is equivalent to separating variance into two components, within the uncontrolled manifold (UCM) (Scholz and Schöner, 1999) and orthogonal to the UCM. Negative *B* values correspond to V_UCM_ > V_ORT_, which is typically interpreted as a sign of synergy stabilizing the resultant variables (Latash et al., 2001). We performed similar analyses of *B* and *R_Z_*; in fact, all the significant effects were the same for the two parameters of the linear regressions (see Results).

**Figure 1:**
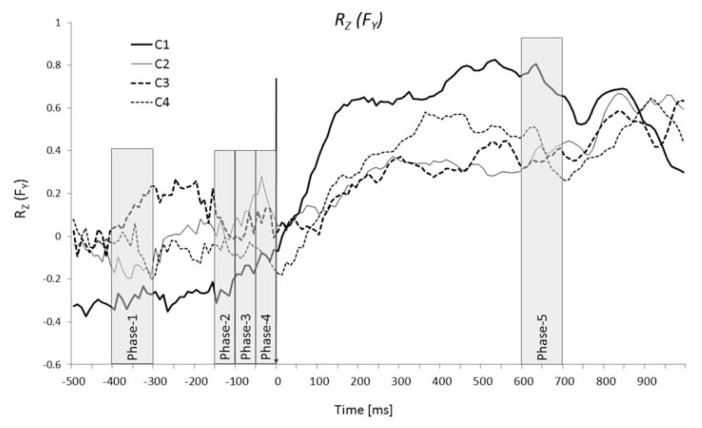
Exemplary plot of mean values of correlation coefficients (Rz) for F_Y_ in different conditions (C1 - one subject, C2 - no contact, C3 - haptic touch, C4 – strong coupling). The designated Phases (1–5) selected for statistical analysis are shown

For analysis of anticipatory synergy adjustments (ASAs) (Klous et al., 2011; Krishnan et al., 2011) to jumping, four time windows were selected, and the averaged within each time window *B* and *R_Z_* values were compared. The time windows were between −300 and −400 ms prior to T_0_ (steady state), − 150 ms to −100 ms, −100 ms to −50 ms, and −50 ms to T_0_. *R_Z_* and *B* values were also averaged within the time interval from +600 ms to +700 ms after T_0_ to represent the movement phase prior to the take-off (Figure 1).

### Statistical analysis

The data are presented in the text and figures as means with standard errors of the mean unless stated otherwise. To test the main hypotheses, we performed the following comparisons. First, we tested whether there was a consistent positive or negative co-variation between the force/moment components recorded by the two force plates during the steady state prior to the jump initiation. For this purpose a single-factor ANOVA with the factor *Condition* (four levels, C1 to C4) was used to compare the *R*-values to zero. Second, we checked whether there was a consistent change between the steady state and movement in *R_Z_* and *B* values. This was done using a two-factor ANOVA with the factors *Conditions* and *Phase* (levels: Phase-1 and Phase-5, see Figure 1). Finally, we explored changes in the force/moment co-variation during the 200 ms time interval prior to T_0_ (ASAs) for the C1 condition only. In other conditions, no consistent ASAs were observed. Thus, a one-way ANOVA with the factor *Phase* (four levels, Phase-1, Phase-2, Phase-3 and Phase-4) was used. The Tukey’s HSD *post-hoc* comparisons were used when needed. We used the Shapiro-Wilk test to check the data for normality and the Levene’s test was used to assess the equality of variances. The significance level *p* was set at 0.05.

## Results

### General patterns of forces and moment

**Figure 2:**
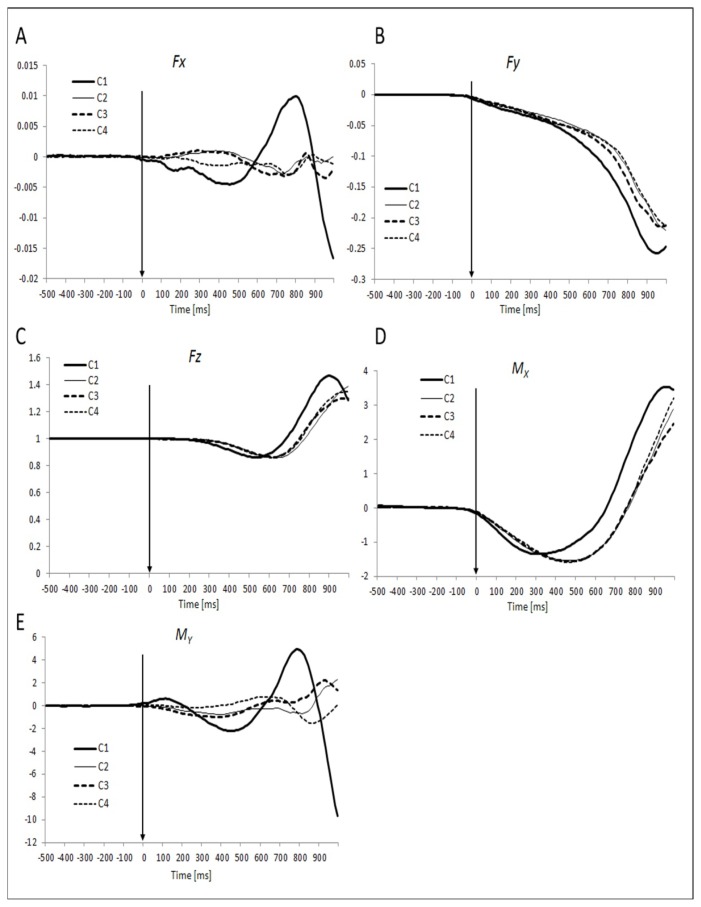
Mean time profiles of forces (Fx, Fy, Fz) and moments of forces (Mx, My) aligned to movement initiation time (T_0_) in different conditions (C1 - one subject, C2- no contact, C3 - haptic touch, C4 – strong coupling)

The force and moment trajectories were consistent across subjects and conditions. The kinetic time profiles for subjects who jumped alone (C1) differed most from the other conditions. This was especially true for the force in the medio-lateral direction (*F_X_*) and moment about the horizontal axis in the anterior-posterior direction (*M_Y_*). In particular, the change of *F_X_* over the analyzed time period was much bigger and showed a different pattern in C1 as compared to the other conditions (Figure 2A). On the other hand, the absolute magnitude of these differences was modest. For *M_Y_* the pattern of changes was consistent across all the conditions, while the magnitude of the changes was much bigger in C1 (Figure 2E). Consistent features of force patterns across conditions included a drop in *F_Y_* after T_0_ and an increase in *F_Z_* about 200 ms after T_0_ (Figures 2B and 2C). The *M_X_* data showed an initial drop immediately after T_0_ followed by a consistent rise across all conditions (Figure 1D). After alignment by action initiation time, the take-off time in C1 (when subjects were jumping alone) was about 150 ms earlier than in conditions C2–C4 (Figure 2).

### Analysis of force-stabilizing synergies

**Figure 3:**
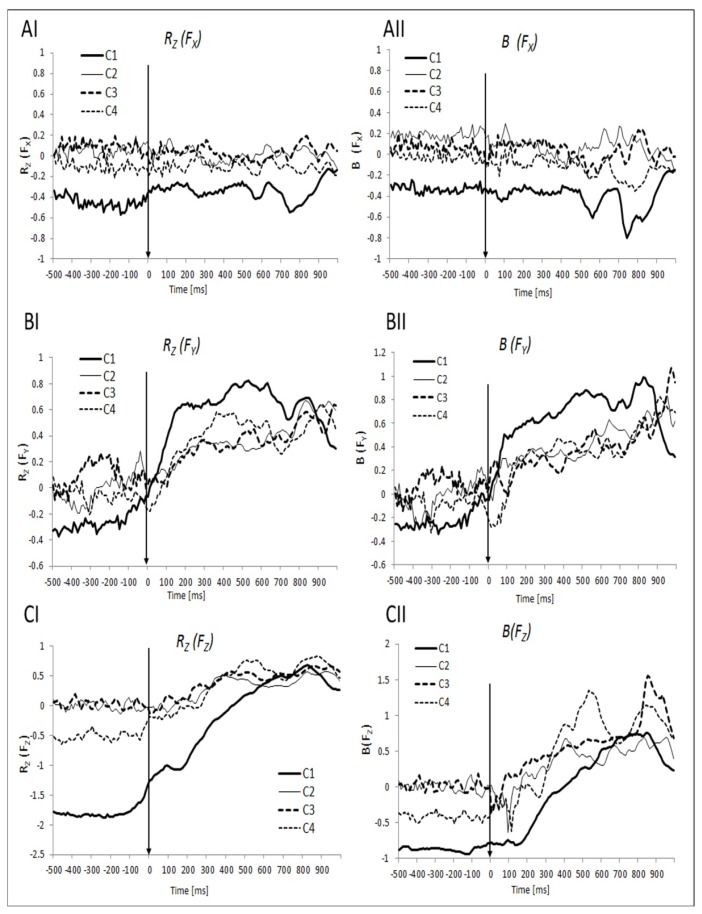
Mean Z-transformed correlation coefficients (R_Z_) and regression coefficients (B) for forces (Fx, Fy, Fz) in different conditions (C1 – one-person; C2- two-persons, no contact; C3 – two-persons, haptic touch; and C4 – two-persons, strong coupling)

When the subjects performed the jumps independently (condition C1), the inter-trial distribution of data from the two force platforms suggested synergies stabilizing the three total force components: Namely, all three force components recorded by the two platforms showed predominantly negative correlations across trials during Phase-1 (steady state) (Figure 3, solid traces). The negative correlation was particularly strong for the vertical force component (*F_Z_*). In the other three conditions with two-person jumping, the correlation coefficients during the steady state were close to zero with the notable exception of the negative correlation coefficient for *F_Z_* in condition C4 (strong contact between the subjects) (Figure 3, panels C).

Overall, *R-values* for *F_X_* showed minimal changes during the jump, while the correlation coefficients for the *F_Y_* and *F_Z_* changed from a predominantly negative to positive. These findings were confirmed with a two-way ANOVA, *Condition* × *Phase,* on Z-transformed *R* (*R_Z_*) for each of the force components. The ANOVAs showed main effects of *Condition* (4 levels) for *F_X_* (F(3,27) = 12.94, *p* < .001) and *Fz* (F(3,27) = 37.36, *p* < .001), and a main effect of *Phase* (2 levels – Phase-1 and Phase-5) for *F_Y_* (F(1,9) = 46.41, *p* < .001) and *F_Z_* (F(1,9) = 136.76, *p*<.001) only. The post-hoc comparisons showed that, for *F_X_*, there was no difference between Phases 1 and 5 in all the conditions.

**Figure 4:**
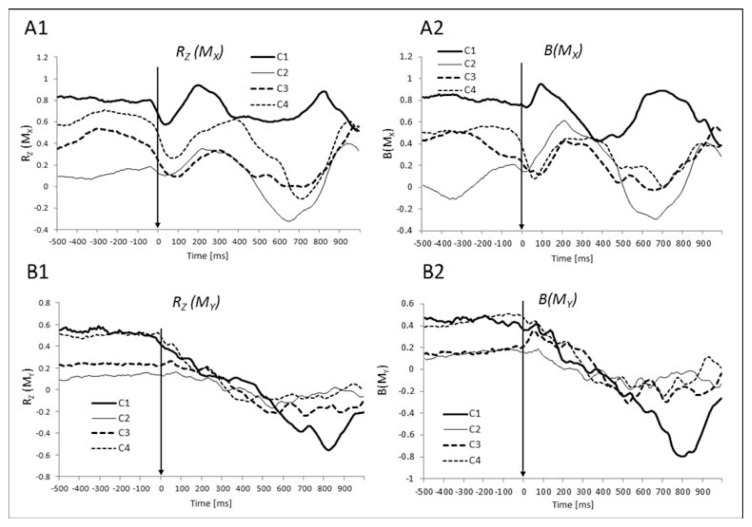
Mean Z-transformed correlation coefficients (R_Z_) and regression coefficients (B) for moments of forces (M_X_, M_Y_) aligned by the movement initiation time (T_0_) across conditions (C1–C4)

For the two moment variables, in the steady state (Phase-1), strong positive correlations were present for *M_X_* in conditions C1 and C4, while they were weaker in condition C3 (Figure 4A1). Similarly, for *M_Y_*, there was a strong positive correlation in conditions C1 and C4 and a weak positive correlation in condition C3. No significant correlations were observed in condition C2. For *M_Y_* the values of *R_Z_* changed from positive to negative correlation with movement progression (Phase-5) in all conditions. This was particularly pronounced in C1. The values of *R_Z_* in condition C1 (solid thick line) and C4 (thin dotted line) for *My* (Figure 4A1) were close to each other in the steady state, while these values were much lower in the other two conditions, C2 and C3. Two-way ANOVA with factors *Condition* (4 levels) and *Phase* (2 levels, Phase-1 and Phase-5) conducted on *R_Z_* for *M_X_* and *M_Y_*, showed a significant main effect of *Condition* (F(3,27) = 37.6, *p* < .001) and *Phase* (F(1,9) = 127.2, *p* < .001) for *M_X_*. For *M_Y_*, there was a significant main effect of *Condition* (F(3,27) = 11.07, *p* < .01) only. The post-hoc comparisons showed that for *M_X_* there was a significant difference between Phase-1 and Phase-5 in all conditions except for condition C1. It is important to state that for *M_Y_* only in condition C1 the difference was statistically significant.

The results also showed significant differences between the values of *R_Z_* for *M_X_* and *M_Y_* in Phase-1 between all conditions except for C1 vs. C4 and C3 vs. C4. The interactions (*Phase* × *Condition*) were significant both for *M_X_* (F(3,27) = 2.98, *p* < .048) and *M_Y_* (F(3,27) = 11.27, *p* < .001). For *M_X_* the change between Phase-1 and Phas-5 was not very distinct, especially in condition C1. The conditions C2–C4 influenced the magnitude of change more than C1. For *M_Y_*, however, the situation was different: C1 showed most significant change between Phase-1 and Phase-5 from positive to less positive. In all other conditions this dependence was inverted and less pronounced.

The time profiles of the regression coefficient *B,* were qualitatively similar to those of *R_Z_* across all the force and moment analyses (Figures 3 and 4, panels II). The statistical effects were similar for *R_Z_* and *B*.

### Analysis of anticipatory synergy adjustments

Synergies stabilizing two components of the force vector showed changes prior to T_0_, i.e., anticipatory synergy adjustments (APAs). The magnitude of the correlation coefficient *R* showed a drop prior to T_0_ for both *F_Y_* and *F_Z_* (Figure 3BI and Figure 3CI). No such changes were observed in the force orthogonal to the direction of jumping (*F_X_*) (Figure 3AI).

**Figure 5:**
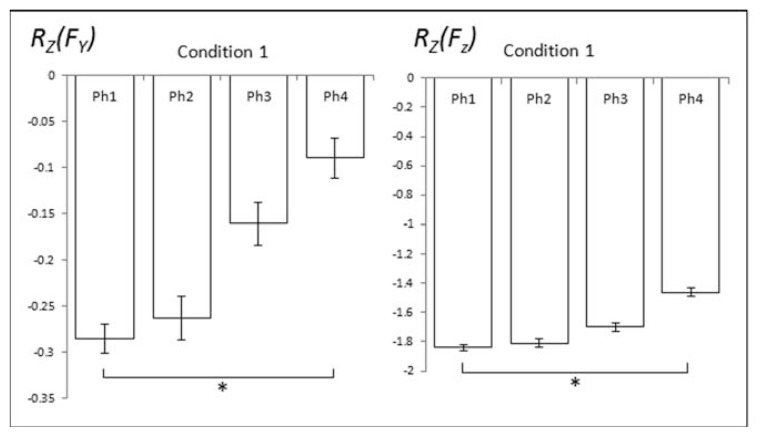
Changes in Z-transformed correlation coefficients (R_Z_) prior to time zero (T_0_) for F_Y_ and F_Z_. The error bars show standard errors across subjects

To explore the APAs, *R_Z_* values were analyzed within four time intervals prior to T_0_ in each condition and compared to the steady state (Phase-1). This was done for all force and moment variables. Significant changes in *R_Z_* (ASAs) prior to T_0_ were seen for *F_Y_* and F_Z_ in condition C1 only (Figure 5). The one-way ANOVA (*Phase*) showed a significant main effect of *Phase* for *F_Y_* (F(3,27) = 3.06), *p* < .05) and for *F_Z_* (F(3,27) = 10.52, *p* < .001). Pairwise contrasts showed significant differences between Phases 1 and 4 for *F_Y_* and *F_Z_* (Figure 5).

## Discussion

With respect to Hypothesis-1 (synergies in one-person conditions, C1), the results provided support for two-leg synergies stabilizing the three force variables, but not for the moment variables. Indeed, during the steady state, the three force variables recorded by the two force platforms in condition C1 showed a negative correlation across repetitive trials; these correlations were particularly strong for F_Z_. This is not surprising given that the sum of the two vertical ground reaction forces had to be very close to the weight of the person during quiet standing. For the two moment variables, positive correlations were observed, suggesting that there were no moment-stabilizing synergies.

When the jumping task was performed by two persons, the two force variables showed no consistent negative correlations during the steady state in support of Hypothesis-2; the strong-contact condition (condition C4) was the only one to show results that were closer to those in the one-person condition. For moment variables, there was a consistent trend towards weaker positive correlations for the two-person conditions; these correlations all but disappear when the two subjects jumped without any physical contact with each other (condition C2). As far as Hypothesis-3 is concerned, few consistent ASAs were seen in one-person trials, and only for force variables. No ASAs were observed in two-person trials.

While we did not formulate any specific hypotheses with respect to possible changes in the force/moment stabilizing synergies during the jump execution (after time zero), there were consistent changes in the correlation coefficients suggesting loss of stabilization of some of the resultant variables (for example, F_Y_ and F_Z_) and the emergence of stabilization of other variables (for example, M_Y_). Similar changes were also seen in two-person tasks. Further, we discuss implications of the obtained results for the different phases during jumping, such as an initial steady state, preparing to and execution of jumping. We also discuss the potential role of different mechanical and sensory factors in the inter-personal coordination of jumping.

### One-person synergies during standing and jumping

Within this study, we accepted a definition of synergy developed within the principle of abundance (Gelfand and Latash, 1998; Latash, 2012). According to this approach, no degrees-of-freedom (DOFs) are eliminated during movements by apparently redundant (actually, abundant) systems of effectors, but the DOFs are organized in a task-specific way to ensure stability of the action in task-specific directions within the redundant space of elemental variables (Schoner, 1995). Two methods have been used recently to estimate the stability of a multi-element system, application of small perturbations and analysis of variance across repetitive trials (Latash et al., 2007; Wilhelm et al., 2013; Zhou et al., 2014). The latter method was developed within the framework of the uncontrolled manifold (UCM) hypothesis (Scholz and Schöner, 1999). Within this approach inter-trial variance in the space of elemental variables is quantified in two spaces computed with respect to performance variables that may be stabilized by synergy. The first sub-space (UCM) corresponds to no changes in the selected performance variables, while this variable changes within the second space (orthogonal to the UCM, ORT). If variance per DOF within the first space (V_UCM_) is larger than in the second (V_ORT_), a conclusion is drawn that synergy stabilizes the performance variable.

**Figure 6:**
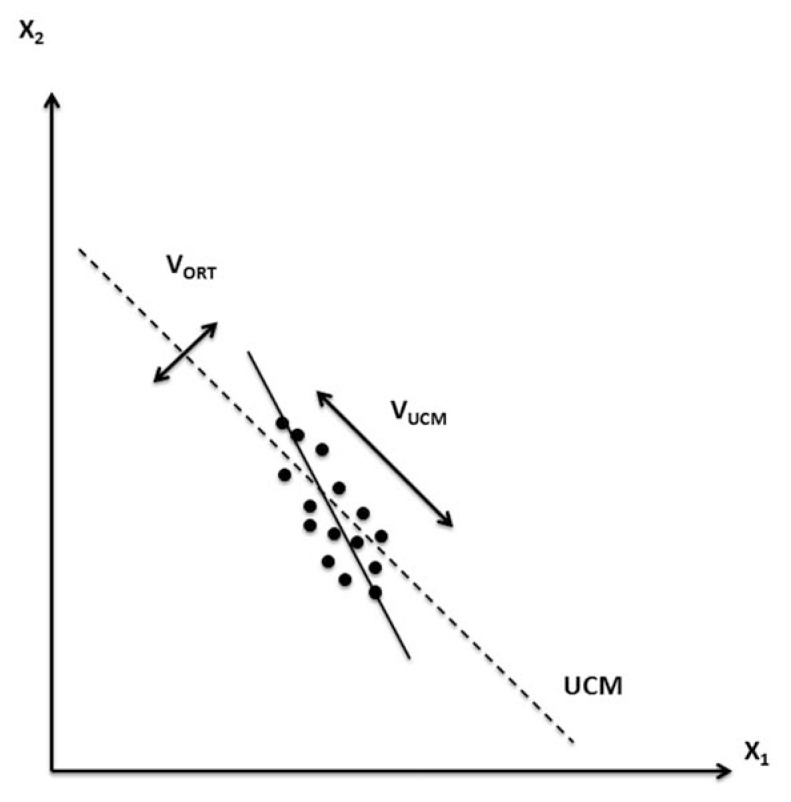
Two elements participate in the task of producing certain magnitude of their sum: X_1_ + X_2_ = C. The space of solutions (the uncontrolled manifold, UCM) is shown with the slanted dashed line. Note that the data cloud across trials is elongated along the UCM; as a result, variance along the UCM (V_UCM_) is larger than variance along the orthogonal direction (V_ORT_). Note that linear regression analysis across the data points has to produce a significant negative correlation (the regression line is shown as a thick slanted line)

When only two variables contribute to a performance variable, analysis can be simplified. Figure 6 illustrates an experiment when the task is to produce a certain value of the sum of two elemental variables: X_1_ + X_2_ = C. If across trials, the data points form an ellipse elongated along the UCM (the slanted dashed line), V_UCM_ > V_ORT_. The same conclusion can be drawn if there is a negative correlation between X_1_ and X_2_ across trials. Hence, we used analysis of the inter-trial correlations between matched pairs of force/moment variables measured by the two force platforms to explore synergies that might stabilize the corresponding resultant variables.

In one-person trials (condition C1), one could expect a strong negative correlation between the two vertical ground reaction force variables (F_Z_), which was observed in the experiment (Figure 3). Indeed, when the person stands quietly, the sum of the two F_Z_ variables has to be close at all times to the weight of the person (a constant, similar to the example in Figure 6). There were also relatively weak correlations between the other two pairs of variables. This result may also be viewed as predictable: although both F_X_ and F_Y_ show changes during the natural postural sway, they change sign frequently (Duarte and Zatsiorsky, 1999) such that the total momentum applied to the body stays about zero.

In contrast, the moments recorded by the two platforms showed strong positive correlations (Figure 4). Note that during postural sway, the coordinate of the center of pressure (COP) shows significant deviations from the original “most comfortable” coordinate and may stay at those new locations for a relatively long time (Duarte and Zatsiorsky, 1999). Thus, in different trials, data points were likely to correspond to different resultant moments about both horizontal axes, M_X_ and M_Y_. The positive correlations between the moment variables recorded by the two force platforms suggest that both feet contributed symmetrically to the resultant moment shifts in both directions.

During the jump execution, two force variables (F_Y_ and F_Z_) showed a switch from negative to positive correlations, while one of the moment variables (M_Y_) switched from a positive to a negative correlation. To interpret these results, we first refer to a series of experimental and theoretical studies (Friedman et al., 2009; Goodman et al., 2005) indicating that V_UCM_ shows close to proportional changes with the magnitude of the performance variable with respect to which the analysis is performed; in contrast, V_ORT_ shows changes with the derivative of the performance variable. During the jump execution, the subjects had to produce quick changes in the resultant F_Z_ and F_Y_ (to generate vertical and horizontal components of the propulsive force), while F_X_ (in the medio-lateral direction) had to be kept close to zero. These quick changes were expected to lead to a switch from the inequality V_UCM_ > V_ORT_ to V_ORT_ > V_UCM_ (Olafsdottir et al., 2005; Sarabon et al., 2013) and, consequently, to a switch from a negative to a positive correlation between the corresponding pairs of variables.

As far as M_Y_ changes are concerned, the resultant M_Y_ had to be kept close to zero to avoid major deviations of the body in the medio-lateral direction (it oscillated about zero, Figure 2E). Given the large changes in the force variables, in particular in F_Z_, large variations in the contributions to M_Y_ from the individual feet could be expected. This resulted in strong synergy stabilizing M_Y_ reflected in the negative correlations between the elemental M_Y_ variables.

### Two-person synergies

A number of studies of two-person synergies have been performed within the dynamic systems approach to movement studies; they used a relative phase of two actions as the performance variable that could be stabilized by adjustments of actions by the individual actors (Fine and Amazeen, 2011; Fine et al., 2013; Riley et al., 2011). In those studies, it was shown that people acting in dyads exhibited similar patterns of limb coordination to those in a single person coordinating multiple limbs (Fine and Amazeen, 2011; Fine et al., 2013; Schmidt et al., 1990). Furthermore, the two persons watched each other while performing various tasks. Our experiment explored this phenomenon by varying the nature and strength of coupling between the two persons. Indeed, when two persons were jumping together without any mechanical contact, they could see each other with peripheral vision (they focused on the target line on the floor), and they were explicitly asked to synchronize their jumps (condition C2). In condition C3, haptic contact between the two actors was provided, while in condition C4 they had strong mechanical contact.

During the steady-state, we have evidence for two-person synergies (negative correlations between elemental force variables) in condition C4 only. In particular, when each person placed one of the arms on the shoulder of the second person, a significant negative correlation between the elemental F_Z_ variables was observed. This correlation was still lower than the one in condition C1. With respect to moment variables, providing a haptic connection between the two persons was sufficient to induce a positive correlation between the elemental moment variables, and this correlation became stronger (closer to that observed in C1) during the strong-coupling condition (C4). As discussed earlier, the positive correlation between the moment variables recorded by the individual force platforms, both for M_X_ and M_Y_, during one-person trials likely reflected COP migration with sway.

Several studies have shown coupling between motor variables in postural tasks, observed when the two participants talked to each other, but not when they stood quietly (Shockley et al., 2002; Stoffregen et al., 2009; Strang et al., 2014). We also observed no significant coupling between the signals from the two platforms in condition C2, when the two subjects stood side-by-side without any additional contact. The results in C3 show that inter-personal sway coupling may be induced by haptic contact. Indeed, these results suggest that the two subjects tended to show similar inter-trial deviations of M_X_ and M_Y_ from their “comfortable stance” values. In other words, their sway in both anterior-posterior and medio-lateral directions was coupled. Haptic information has been shown in many studies to lead to significant sway modulation, e.g., the reduction of sway in the presence of a light finger touch to a stationary object (Jeka and Lackner, 1994). The motion of the touched surface has been shown to entrain sway (Jeka et al., 1998). Our results show that haptic information is also efficient when a person touches an object held by another person.

While there were significant differences across two-person conditions during the steady state for some force/moment variables, these differences tended to disappear during the jump performance. This is clear for both force (Figure 3) and moment (Figure 4) variables. This observation suggests that mechanical requirements of the task overpowered the differences in inter-personal coupling observed in the steady-state across conditions.

### Changes in stability prior to action initiation

As mentioned earlier, the amount of variance leading to a change in a selected performance variable (V_ORT_) changes with the time derivative of that variable (Goodman et al., 2005). Thus, typically, if a performance variable is stabilized by synergy at a steady state, the synergy disappears when the variable is changed quickly. In our experiment, this was seen as the change from a negative to a positive correlation after the action initiation (time zero). Such changes were clearly seen for the two force variables stabilized at the steady state, F_Y_ and F_Z_. They were not observed for the third force variable, force in the medio-lateral direction, F_X_, which showed inconsistent changes during the jump. Actually, a drop in the absolute values of negative correlations could be seen for *F_Y_* and *F_Z_* prior to the action initiation (Figures 3 and 5). These changes cannot be attributed to derivative-dependence of those resultant variables as their changes had not started yet. They represent examples of anticipatory synergy adjustments (ASAs), feed-forward adjustments in strength of synergy stabilizing a variable in anticipation of a planned quick change of that variable (Jae et al., 2005; Klous et al., 2011; Krishnan et al., 2011; Olafsdottir et al., 2005).

ASAs are observed only during predictable quick changes in a performance variable, when the person has time to prepare for the action. For example, when a similar action is performed under the simple reaction time instruction, no APAs are observed (Olafsdottir et al., 2005). When a person performs a jump in a self-paced manner, ASAs are expected for the mechanical variables that will be changed quickly during the jump (components of the propulsive force), and indeed, they were observed in the experiment. When two persons perform the task in parallel, even in conditions with strong contact, they cannot be absolutely certain when the action will start and how it will proceed. As a result, no ASAs were observed in our experiment in two-person conditions, even in condition C4. These observations suggest that, although synergies can be seen in two-person actions, feed-forward adjustments of synergies require the involvement of a single central nervous system.

## Concluding comments

To summarize, we observed the strongest correlations between the pairs of mechanical variables recorded by the two force plates in condition C1, when one person performed the task. This condition was also the only one to show ASAs. These observations suggest that synergies and resulting adjustments are primarily under feed-forward control, which can only be exercised effectively when one central nervous system is in full control of the performance. When the two persons participate in a task, even if multiple sources of sensory information were available between the two central nervous systems, most signs of synergies and consequent adjustments disappeared. Even though some of the earlier studies have showed a positive influence of visual or haptic coupling on inter-personal motor performance, this coupling does not seem to be sufficient for the creation of synergies stabilizing performance variables. Overall, the results of the current study are most directly compatible with the model of synergies based on central back-coupling loops within the central nervous system (Latash et al., 2005); they are less compatible with schemes that rely on sensory feedback.

Our study is not without drawbacks. One important limitation is narrowing the analysis to kinetic variables only. It would be of additional value to analyze muscle activation patterns, something we plan to accomplish in the future. Also, in future studies, we would like to explore the possibility of training subjects in order to create interpersonal synergies. Identification of the emergence of interpersonal synergies in sports that require synchronized actions such as rhythmic gymnastics, dance or synchronized swimming or diving might be beneficial for sports training, facilitating the process of learning of synchronized actions. Development of the methods that would be able to relatively quickly identify interpersonal synergies would be of the essence and might substantially improve the process of sports training. It would also be interesting to test subjects who have already been trained to synchronize their actions with another person.
